# Amino Acid Derivatives of Chlorin-e_6_—A Review

**DOI:** 10.3390/molecules28083479

**Published:** 2023-04-14

**Authors:** Maria da Graça H. Vicente, Kevin M. Smith

**Affiliations:** Department of Chemistry, Louisiana State University, Baton Rouge, LA 70803, USA; vicente@lsu.edu

**Keywords:** chlorin-e_6_, amino acid conjugate, photodynamic therapy, NPe6, Talaporfin, LS-11, Laserphyrin^®^

## Abstract

Details of the structural elucidation of the clinically useful photodynamic therapy sensitizer NPe6 (**15**) are presented. NPe6, also designated as Laserphyrin, Talaporfin, and LS-11, is a second-generation photosensitizer derived from chlorophyll-a, currently used in Japan for the treatment of human lung, esophageal, and brain cancers. After the initial misidentification of the structure of this chlorin-e_6_ aspartic acid conjugate as (**13**), NMR and other synthetic procedures described herein arrived at the correct structure (**15**), confirmed using single crystal X-ray crystallography. Interesting new features of chlorin-e_6_ chemistry (including the intramolecular formation of an anhydride (**24**)) are reported, allowing chemists to regioselectively conjugate amino acids to each available carboxylic acid on positions 13^1^ (formic), 15^2^ (acetic), and 17^3^ (propionic) of chlorin e_6_ (**14**). Cellular investigations of several amino acid conjugates of chlorin-e_6_ revealed that the 13^1^-aspartylchlorin-e_6_ derivative is more phototoxic than its 15^2^- and 17^3^-regioisomers, in part due to its nearly linear molecular conformation.

## 1. Introduction

**Figure 1:**
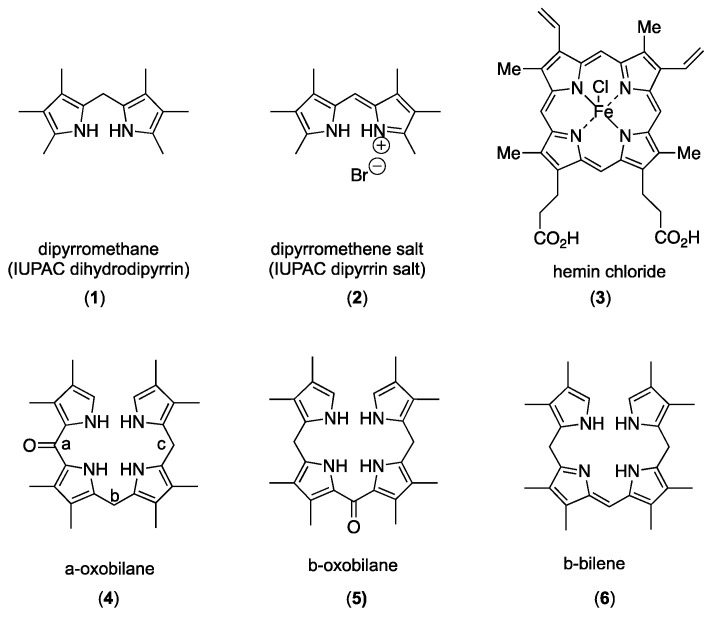
Structures of various porphyrin precursors and hemin chloride (**3**).

In the early 1960s, a new porphyrin research group in Liverpool, UK, under the direction of George W. Kenner F.R.S and Anthony H. Jackson, began to make small steps towards development of new porphyrin syntheses. The field of porphyrin synthesis had mostly been dominated by Hans Fischer’s group in Munich [1,2,3]; the new approach in Liverpool was to use dipyrromethanes (with two pyrroles linked through an sp^3^ carbon) (**1**) rather than the unsaturated dipyrromethene salts (**2**) that had yielded literally dozens of synthetic porphyrins for Fischer’s group, this work culminating in award of the Nobel Prize for Chemistry to Fischer in 1930. The prize was principally for the unambiguous synthesis of the blood pigment hemin (**3**) (Figure 1), though much else had been achieved. Fischer’s group had briefly dabbled with dipyrromethanes, but the heart of their porphyrin synthesis achievements lay in their dipyrromethene approaches. Dipyrromethanes (**1**) are somewhat acid-sensitive molecules that could not survive Fischer’s relatively harsh acid conditions used to forge the porphyrin macrocycle from dipyrromethene salts (**2**). The Liverpool group enjoyed early successes and was able, in the 1960s, to develop new approaches to porphyrins using so-called a-oxobilanes (**4**) [4], b-oxobilanes (**5**) [5], and b-bilenes (**6**) [6].

**Figure 2:**
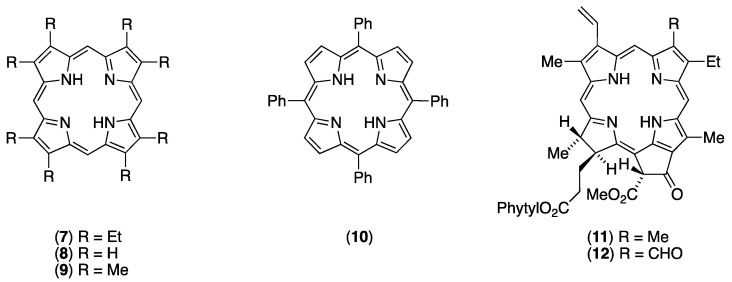
Structures of porphyrins (**7**–**10**) and pheophytins-a and -b.

**Figure 3:**
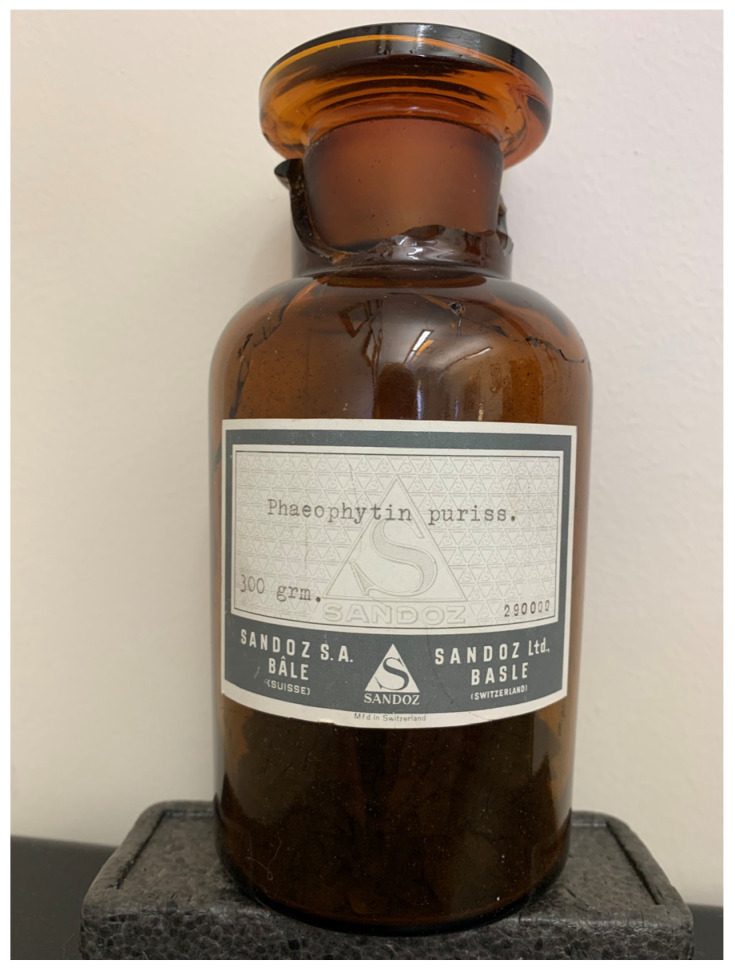
Gift bottle containing “Phaeophytin puriss. 300 grm.” (ca. 1961).

At this point in time, Kenner began interacting personally with Hans Herloff Inhoffen in Braunschweig, Germany. Inhoffen had persuaded one of his former students, a Dr. Pommer, who occupied a high-level position in the German chemical industry, to produce octaethylporphyrin (OEP; **7**) for him on the kilogram scale; this industrial-size gift fed a post-Fischer renaissance in porphyrin chemistry that lasts even to this day. As the choice model porphyrin for future development of porphyrin science, OEP was far more soluble in organic solvents than unsubstituted porphyrin (**8**) and even more than octamethylporphyrin (**9**); it also appeared more “relevant” to natural systems than was *meso*-tetraphenylporphyrin (**10**) (Figure 2). During a visit to Braunschweig, one of the present authors (KMS) benefited from a gift of no less than 20 g of Pommer’s OEP [7]. After one of his 1960–1961 visits to mainland Europe, Kenner returned to Liverpool with a gift: a heavy brown glass bottle labeled “Phaeophytin puriss. 300 grm.” (Figure 3). It is unknown, to the authors at least, if this gift was also another indirect donation to science by Dr. Pommer. His kilograms of OEP were going to keep Inhoffen and his talented senior collaborators in Braunschweig (alphabetically, H. Brockmann, Jr.; J-H. Fuhrhop, A. Gossauer, and H. Scheer) busy for some time into the future, making many new discoveries around and about porphyrin and porphyrinoid chemistry. The pheophytin gift helped the Liverpool group to spread into the chlorophyll research area.

Pheophytins are an approximately 3:1 mixture of magnesium-free chlorophyll-a (**11**) and -b (**12**). In Liverpool, work with the pheophytins resulted in some new, or at least improved, methods for degradation of the chlorophyll chromophore to give pheophorbides, chlorins, rhodins, purpurins, and other exotically named molecules [8]. However, there was a problem. As mentioned above, the pheophytins are a mixture of the -a and -b series, and these needed to be separated to be useful. In those pre-HPLC days, the two series of pigments were usually separated using sucrose gravity column chromatography; the Robert Robinson Laboratories at the University of Liverpool often smelled of caramel for weeks on end due to the uncontrolled drying of kilograms of sucrose on trays in glassware rack ovens. Progress in those days was slow, but improvement was realized when we developed a convenient multi-gram-scale method for separation of the (magnesium-free) -a and -b series chlorophyll derivatives using the “Girard T” reagent. The -a/-b mixture was treated with an excess of Girard’s T reagent in acetic acid/chloroform/methanol to give the unreacted -a component and the quaternary ammonium salt Girard adduct at the -b series aldehyde group. Chromatography of this mixture on deactivated neutral alumina gave a fast first band of pure -a series pigment, the Girard salt adduct being firmly stuck to the alumina at the top of the chromatography column. Dramatic increase in the polarity of the solvent eluted the Girard b-series adduct, which could be converted into pure -b series pigment by treatment with methanol and sulfuric acid. This separation procedure was performed many times on pheophytins (**11**/**12**) at and above the 2.5 g scale, eventually resulting in pure samples of methyl pheophorbide-a and -b [8].

Then, we discovered the existence of *Spirulina* alga, which Wasielewski and Svec [9] had been using as a source of pure chlorophyll-a pigment—and only chlorophyll-a! From UC Davis we purchased oil-barrel-sized quantities of *Spirulina maxima* cheaply from Sosa Texcoco in Mexico. After a while, since *Spirulina* is also a regulated health food, our drums of alga (which were spray-dried on sand in Mexico) were increasingly rejected by the US customs at the border in Laredo, Texas, (and in San Diego, CA, which our supplier tried as a work-around); the drums contained dead mosquitoes and bird droppings admixed with the *Spirulina*. US customs did not respond to our claims that we did not want to eat it. Therefore, we switched to Cyanotech in Hawaii for our alga (*Spirulina pacifica*), and in no time it began arriving via United Airlines and our supplies of chlorin-e_6_ began to build [10]. Currently, there are multiple suppliers of *Spirulina* worldwide.

**Figure 4:**
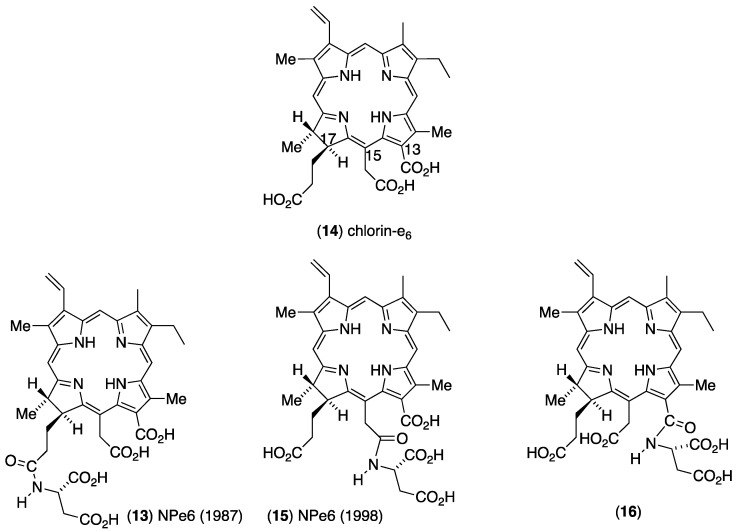
Structures of chlorin-e_6_ (**14**) and the three possible regioisomers (**13**, **15**, **16**) for NPe6.

Chlorin-e_6_ (**14**) itself was shown [11] to be an ineffective photosensitizer for photodynamic therapy, and for this reason its conjugates were investigated (Figure 4). In 1987, a patent was issued to a Utah company named Porphyrin Products for the use of *N*-aspartylchlorin-e_6_ (NPe6, Talaporfin, LS-11, Laserphyrin^®^) in photodynamic therapy [12]. The assigned structure for NPe6 at the time was (**13**), with the aspartyl group attached to the 17^3^-carbonyl; it was prepared by treatment of chlorin-e_6_ (**14**) with aspartic acid, an organic base, and a peptide coupling reagent. The aspartyl residue on the isomerically pure NPe6 product was assigned to the (presumably more reactive) propionic acid side chain. Later, in 1998, the structure of NPe6 was revised to (**15**) [13] (see Section 3).

Given the non-regioselective commercial synthesis of NPe6, there are nominally three possible regioisomers (**13**, **15**, and **16**), that can be formed in the conjugation process (Figure 4). This is because chlorin-e_6_ (**14**) has three carboxylic acid groups, at positions 13^1^ (formic), 15^2^ (acetic), and 17^3^ (propionic), each with presumably different reactivities. We therefore began to focus on the use of chlorin-e_6_ conjugates as photosensitizers for the treatment of tumors. Chlorin-e_6_ (**14**) is a bright grass-green-colored pigment in dichloromethane solution, with an intense long-wavelength absorption maximum at 660 nm; this long-wavelength absorption facilitates photodynamic treatment of larger tumors because low-energy light travels deeper into tissue than does the easily scattered high-energy light required, for example, by the hematoporphyrin mixture Photofrin^®^.

## 2. Photodynamic Therapy Using NPe6

Photodynamic therapy (PDT) is a type of phototherapy that combines a photosensitizer, laser light, and oxygen to selectively destroy targeted cells and tissues [14,15,16]. The activation of the photosensitizer with light produces singlet oxygen and reactive oxygen species (ROS) that cause oxidative damage to cell components such as lipids and proteins, leading to cell death by multiple mechanisms [17]. Additional PDT effects include the shutting down of tumor blood vessels and stimulation of the immune system that leads to anti-tumor immune responses. PDT has advantages over other therapies in that it is minimally invasive, highly localized to light-activated and photosensitizer-containing cells, requires short treatment times, and allows for multiple treatments to be administered with limited adverse long-term side effects to the patient.

Since the approval of Photofrin^®^ as a photosensitizer for PDT by various health organizations around the world, a considerable amount of research has focused on the development of other porphyrin-based drugs with stronger absorbance in the red region of the visible spectrum, where light penetrates deeper through human tissues, and with enhanced tumor-selectivity compared with the first-generation Photofrin^®^ sensitizer. One such compound, NPe6 (**15**), is a second-generation photosensitizer with an intense absorption at 664 nm, with the ability to produce singlet oxygen at high-quantum yield, and faster clearance from skin compared with Photofrin^®^ (the plasma half-life of Talaporfin is about half that of Photofrin^®^). Although chlorin-e_6_ (**14**) also has the ability to preferentially accumulate in tumor tissue, mainly due to the enhanced permeability and retention (EPR) effect, its aspartic acid derivative (**15**), showed increased selectivity for tumor tissues and enhanced phototoxicity, with rapid clearance from normal tissues in pre-clinical trials [18]. This is in part due to its increased affinity towards lipoproteins and endogenous amino acid receptors compared with chlorin-e_6_.

NPe6 was further investigated in pre-clinical trials in the 1980s and 1990s [19,20,21,22]. It was shown that NPe6 binds to albumin and low- and high-density lipoproteins (HDL, LDL), is taken up by tumor cells through endocytosis, accumulates mainly in the lysosomes, and was shown to induce apoptosis, necrosis, and autophagy-associated cell death after light irradiation [23,24,25,26]. Currently, NPe6 has been approved in Japan for the treatment of early and advanced lung cancers [27,28], esophageal cancer [29,30], and intracranial brain tumors [31,32].

In addition to cancer treatment, PDT is also used for the treatment of a variety of infectious diseases caused by bacteria and viruses due to its activity against a wide range of microorganisms, including Gram-positive and Gram-negative bacteria [33]. One advantage of antimicrobial PDT versus antibiotic therapies is the lack of resistance to singlet oxygen, the main cytotoxic agent in PDT. The release of singlet oxygen leads to membrane and DNA damage that leads to the death of the pathogen. Among the photosensitizers investigated in antimicrobial PDT, positively charged chlorin-e_6_ derivatives have shown activity against multiple pathogenic bacteria and fungi due to their more efficient binding to their negatively charged outer membranes.

## 3. Structure Elucidation of NPe6

The revelation of Gomi et al. [13] in 1998 that NPe6 was in fact the 15^2^-aspartyl conjugate (**15**) of chlorin-e_6_ (**14**) failed to gather much support; indeed, it was largely ignored. The manufacturer/distributor continued to use the 17^3^-conjugate structure (**13**) in its literature and on its sample labels. Some clinicians questioned, at conferences, whether the structure they were using in their publications was correct. Gomi et al. [13] used NMR spectroscopy and some chemical degradation studies to settle upon their proposed structure (**15**). To some it seemed that aspartyl conjugation at a sterically congested 15-side chain was far less likely than at the rotation-free uncongested 17^3^-position, and presumably found structure (**15**) hard to believe.

These uncertainties and personal requests caused us to obtain an authentic sample of NPe6 from the distributor, prepare the tetramethyl ester using diazomethane, and then attempt to obtain crystals suitable for single-crystal X-ray diffraction. After many months and some hundreds of individual crystallization experiments, nothing of use materialized. We were then forced to establish a synthetic program to settle the matter. It was decided to unambiguously synthesize all three (if necessary) possible NPe6 regioisomers (**13**, **15**, **16**) and then, as tetramethyl esters, compare them with an authentic sample from the manufacturer.

### 3.1. Unambiguous Partial Synthesis of 17^3^-Aspartylchlorin-e_6_ (***13***)

**Scheme 1:**
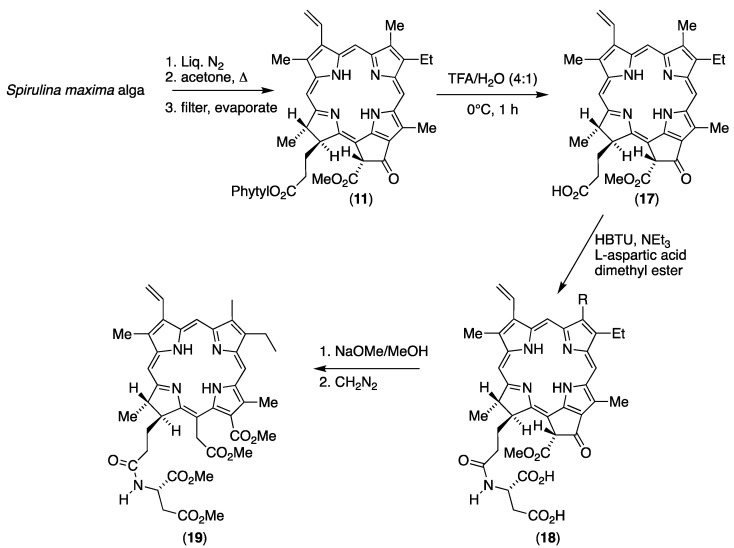
Synthesis of 17^3^-aspartylchlorin-e_6_ tetramethyl ester (**19**).

Pheophytin-a (**11**) was extracted from *Spirulina maxima* alga using the published methodology [11]. Brief treatment with TFA/H_2_O at 0 °C cleaved the phytyl ester to give pheophorbide-a (**17**) (Scheme 1) [34]. Conjugation with L-aspartic acid dimethyl ester in the presence of HBTU and NEt_3_ gave 17^3^-aspartylpheophorbide-a (**18**), which was subjected to isocyclic ring-cleavage in methoxide/methanol [8] to give (**13**) as its tetramethyl ester (**19**). As a precaution against accidental hydrolyses during the methoxide treatment, the product was treated with excess diazomethane before crystallization.

**Figure 5:**
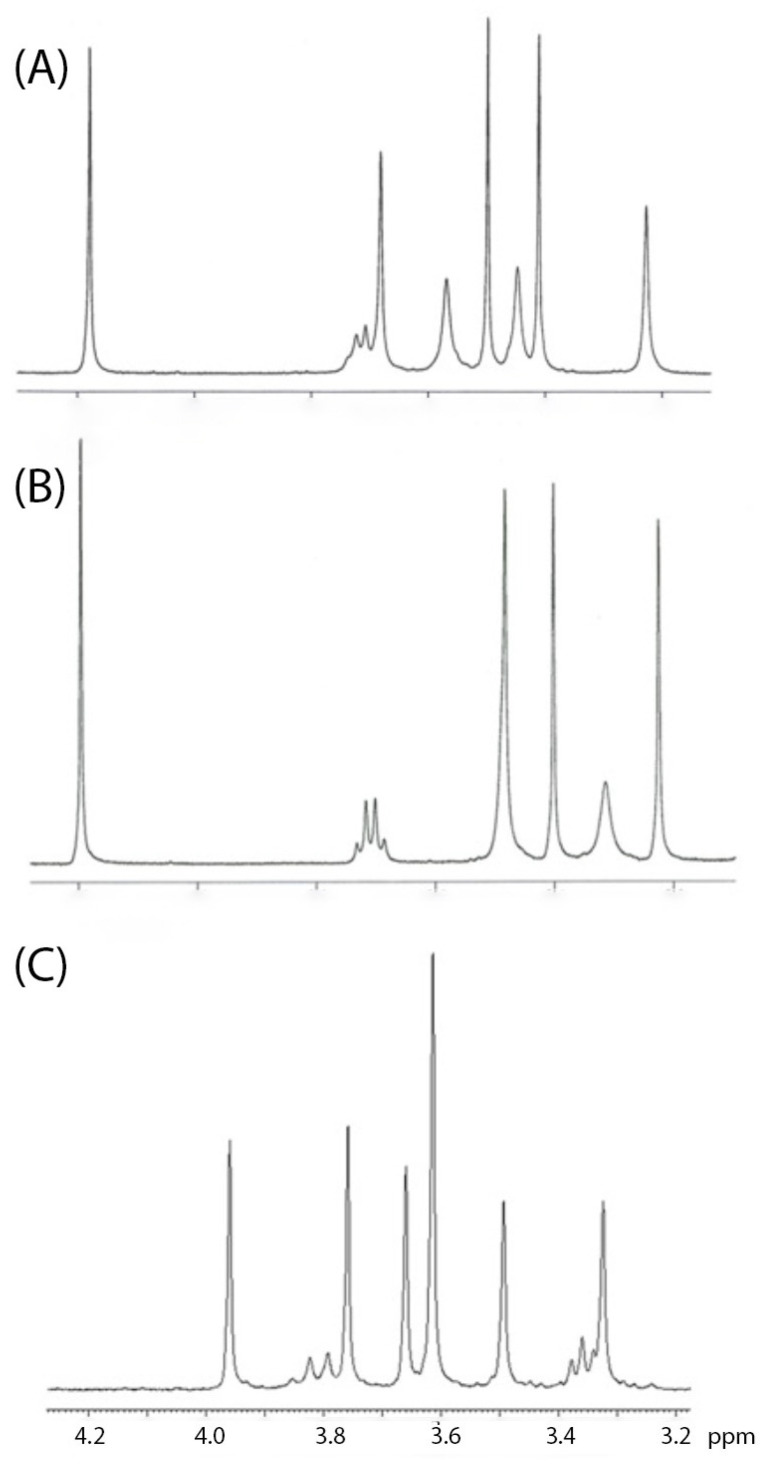
Proton NMR spectra (360 MHz, 3.2–4.2 ppm region, in CDCl_3_) of (**A**) 17^3^-aspartylchlorin-e_6_ tetramethyl ester (**19**), (**B**) authentic NPe6 tetramethyl ester (15^2^ regioisomer, (**23**)), and (**C**) 13^1^-aspartylchlorin-e_6_ tetramethyl ester (**22**). The signals at ~4.2 ppm in (**A**,**B**) correspond to the methyl ester of the 13^1^ substituent.

Proton NMR spectroscopy (Figure 5) showed that the spectrum of synthetic tetramethyl ester (**19**) was significantly different from that of an authentic sample of NPe6 after treatment with diazomethane to convert it into its tetramethyl ester.

### 3.2. Unambiguous Partial Synthesis of 13^1^-Aspartylchlorin-e_6_ (***16***)

**Scheme 2:**
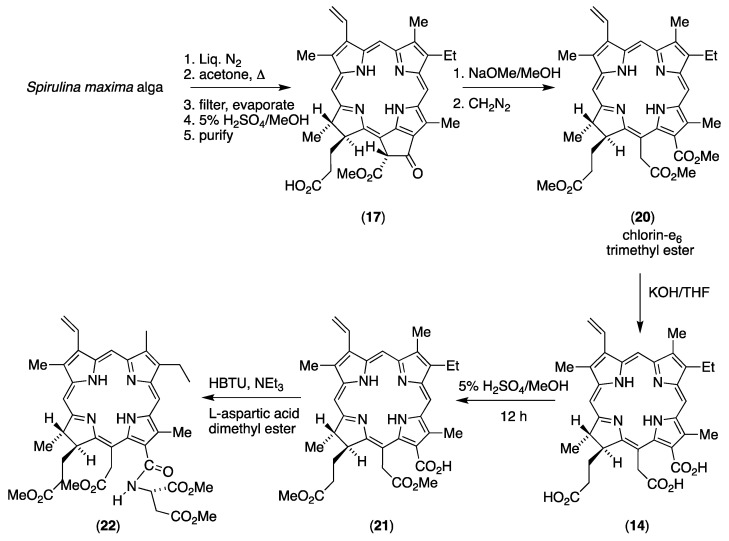
Synthesis of 13^1^-aspartylchlorin-e_6_ tetramethyl ester (**22**).

Once again, *Spirulina maxima* alga was extracted to yield pheophorbide-a (**17**); this was treated with methoxide in methanol, followed by diazomethane to give chlorin-e_6_ trimethyl ester (**20**), which was thoroughly purified via column chromatography before being hydrolyzed to chlorin-e_6_ (**14**) using potassium hydroxide in tetrahydrofuran [34]. At this point, we employed an observation we made in Liverpool in 1974 [35]; if chlorin-e_6_ (**14**) is esterified under acidic conditions (5% H_2_SO_4_/MeOH), esterification of the nuclear carboxylic acid at position-13 is selectively inhibited due to protonation of the two imine-type nitrogen atoms of the chlorin macrocycle [36]. In this way, a 95% yield of the chlorin-e_6_ dimethyl ester monocarboxylic acid (**21**) was obtained from this partial esterification procedure (Scheme 2). Treatment of (**21**) with L-aspartic acid dimethyl ester in the presence of HBTU and NEt_3_ afforded the 13^1^-aspartyl conjugate (**22**) as its tetramethyl ester. Once again, proton NMR spectroscopy of the tetramethyl esters (Figure 5) revealed that authentic NPe6 tetramethyl ester was not identical with the tetramethyl ester of the 13^1^-regioisomer. NPe6 therefore had to be (**15**) as proposed by Gomi et al. [13]. No sooner had we confirmed the structure of NPe6 as (**15**) to our own satisfaction, and only then, did crystals begin to appear in our X-ray sample tubes!

### 3.3. X-ray Crystal Structure of Authentic NPe6 Tetramethyl Ester (***23***)

**Figure 6:**
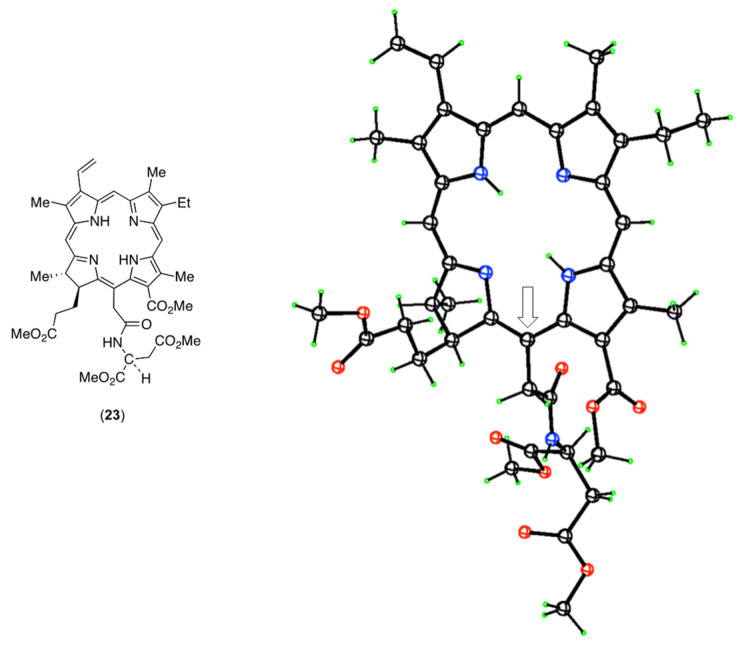
X-ray structure of authentic NPe6 tetramethyl ester (**23**).

Figure 6 shows the X-ray structure of authentic NPe6 tetramethyl ester (**23**) [34]. As proposed by Gomi et al. [13], the structure of NPe6 was definitively established as 15^2^-mono-L-aspartylchlorin-e_6_ (**15**).

With the knowledge that the aspartyl residue is indeed conjugated to the 15^2^-position, the question next raised was “Why?” This regiochemistry was not expected and there was some disbelief even on the part of the manufacturer/distributor when the 15^2^-conjugate structure was proposed. Once one knows with certainty the identity of the product, deducing why this is so becomes significantly easier.

### 3.4. An Anhydride Intermediate (***24***) in the Formation of NPe6

**Scheme 3:**
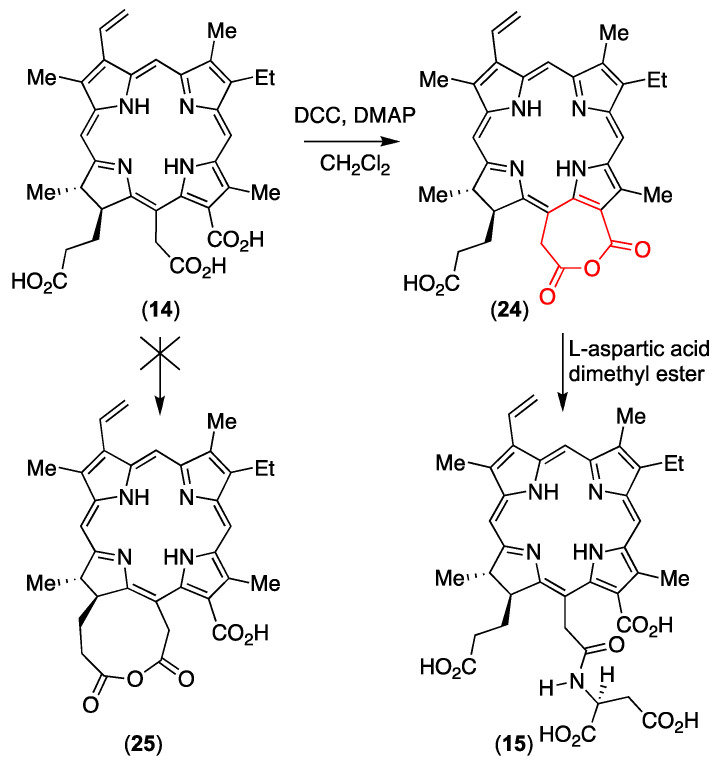
Proposed pathway for synthesis of NPe6 (**15**) via the anhydride (**24**).

**Figure 7:**
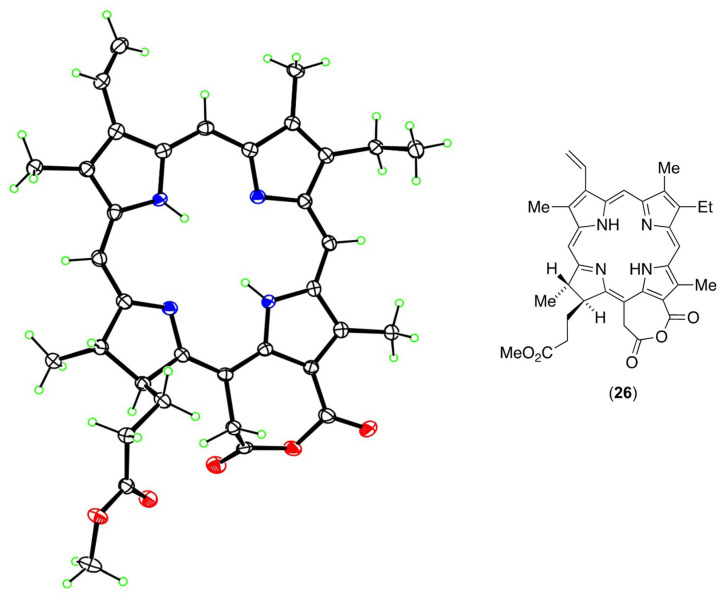
X-ray structure of anhydride methyl ester (**26**).

The most likely explanation for the formation of the 15^2^-conjugate seemed to be that an anhydride (**24**) was formed between the chorin-e_6_ 15^2^ and 13^1^ carboxylic acid groups [34]. This was more likely than a larger ring anhydride (**25**) between the 17^3^ and 15^2^ acids (Scheme 3). The aspartic acid nitrogen atom would then undergo nucleophilic attack upon the aliphatic side of the anhydride (**24**) to produce the 15^2^ conjugate (**15**). To investigate this possibility, chlorin-e_6_ was treated with the DCC coupling reagent and DMAP, but in the *absence* of aspartic acid. The color of the solution turned from green to purple. We were not unaware of the fact that Hans Fischer [3] named certain anhydride derivatives of chlorophylls as “purpurins” because they are indeed purple in color! The purple material was isolated, treated with diazomethane, and crystallized to give the methyl ester (**26**). It was then investigated using single-crystal X-ray diffraction. The structure is shown in Figure 7, and the intermediacy of the anhydride in these conjugation reactions was firmly established [37]. 

### 3.5. The Coup de Grace—Synthesis of NPe6 Tetramethyl Ester (***23***) from the Anhydride (***26***)

A final task was to stablish that the anhydride (**24**) did indeed react to give a 15^2^-conjugate (**15**) when treated with aspartic acid. Thus, anhydride methyl ester (**26**) was treated with aspartic acid dimethyl ester and DMAP, smoothly giving a product identical with authentic NPe6 tetramethyl ester (**23**) [37]. These results clearly established that the pathway from chlorin-e_6_ (**14**) to give the unexpected regioisomer (**15**) involved the intermediacy of the anhydride (**24**).

**Figure 8:**
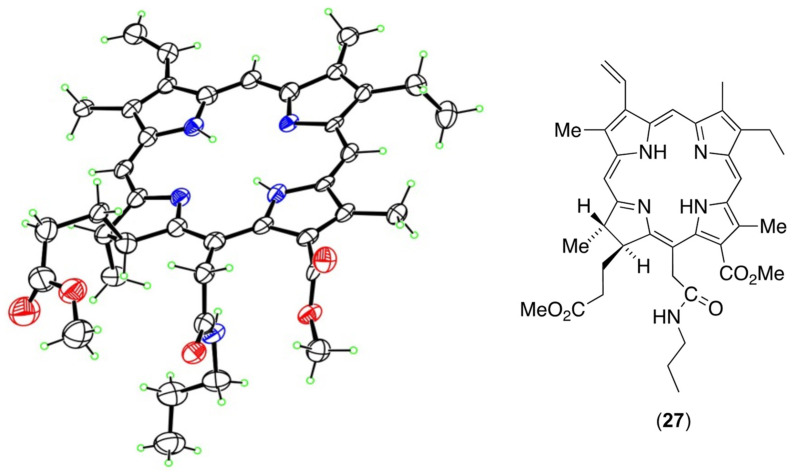
X-ray structure of 15^2^-propylamide (**27**).

Reactions of (**26**) with a wide variety of nucleophiles (ethoxide, propylamine, isopropylamine, ethanolamine, p-tolylthiolate, phenoxide, isobutoxide, and benzyloxide) all yielded the 15^2^-conjugates, with several of these structures (e.g., Figure 8) being confirmed by single-crystal X-ray structures [37]. In all these reactions, a clear color change from green (chlorin-e_6_) to purple (anhydride) upon addition of peptide coupling reagent and base, and then back to green (chlorin-e_6_) after the addition of nucleophile, was observed; reaction progress could simply by tracked using the naked eye.

## 4. Phototoxicity of NPe6 Amino Acid Conjugates

Using the strategies described above for the synthesis of 17^3^-, 15^2^-, and 13^1^-aspartylchlorin-e_6_, several amino acid conjugates of chlorin-e_6_ were investigated for their cytotoxicity, cellular uptake, and intracellular distribution using human HEp2 cells [36,38]. The dark- and photo-toxicity of these derivatives are shown in Table 1. Interestingly, the phototoxicity depends significantly on the position of conjugation on chlorin-e_6_, rather than the nature of the amino acid or the presence of a centrally chelated Pd(II) ion, with the 13^1^-derivatives being the most phototoxic. This might be due to the different molecular conformations conferred by the 17^3^-, 15^2^-, or 13^1^-substitutions [36]. Indeed, it was shown that the 17^3^-derivatives assume a L-shape conformation with the 17^3^-amino acid positioned nearly perpendicular to the macrocyclic plane, whereas the 15^2^-amino acid forms approximately a 120° angle and the 13^1^-amino acid nearly a 180° angle with the macrocyclic plane. The extended, and in the case of the 13^1^-derivatives, almost linear conformations of the amino acid chlorin-e_6_ conjugates likely favors binding to biomolecules, enhancing their phototoxic effect. In agreement with these results, a 13^1^-cystein derivative of chlorin-e_6_ was reported to display higher phototoxicity compared with its 15^2^-regioisomer [39]. More recently, the 3^1^-hexyloxy derivatives of 15^2^-lysylchlorin-e_6_ and 13^1^-aspartylchlorin-e_6_ were shown to be more potent than NPe6 against B16/F10 tumors in C57BL/6 mice [40].

Among the amino acid derivatives of chlorin-e_6_, the positively charged lysine derivatives were observed to be more efficiently internalized by cells, due to their favorable interactions with the negatively charged plasma membranes. A 17^3^-arginine chlorin-e_6_ derivative was shown to have significantly higher phototoxicity toward K562 myeloid leukemia cells at low light doses compared with chlorin-e_6_ [41]. All amino acid derivatives of chlorin-e_6_ were also found to localize preferentially in the lysosomes and the ER, and to a smaller extent in the Golgi apparatus and mitochondria [36]. 

## 5. Conclusions

Photodynamic therapy is a is minimally invasive modality used worldwide for the treatment of cancers and various other diseases, including antibiotic-resistant microbial infections. It uses a photosensitizing drug, which upon activation with laser light produces singlet oxygen and ROS that destroy targeted cells. NPe6, also known as Talaporfin, LS-11, and Laserphyrin, is a second-generation photosensitizer currently approved in Japan for the photodynamic treatment of lung, esophageal, and brain cancers, including glioblastomas. In the 1980s, 1990s, and early 2000s, the structure of NPe6 was mis-represented as the 17^3^-aspartylchlorin-e_6_ derivative (**13**). However, the systematic synthetic availability of all possible regioisomers of NPe6 (**13**, **15**, **16**), their NMR structural characterization, and, finally, an X-ray crystal analysis of an authentic derivative, unequivocally confirmed the structure of NPe6 as compound (**15**). Several amino acid conjugates of chlorin-e_6_ were then synthesized and evaluated for their photodynamic properties; these studies revealed that the 13^1^-aspartylchlorin-e_6_ is more phototoxic than its 15^2^- and 17^3^-regioisomers, respectively, and displays a higher dark- to phototoxicity ratio (>460). This might be due to the nearly linear molecular conformation of the 13^1^-compound, where the aspartic acid chain extends away from the macrocyclic ring; in the case of the 15^2^- and 17^3^-compounds, the amino acid forms approximate 120° and 90° angles, respectively, with the macrocycle. The almost linear molecular geometry of the 13^1^-aspartylchlorin-e_6_ derivative likely favors its binding to multiple cellular components, enhancing its phototoxic effect compared to its 15^2^- and 17^3^-regioisomers.

## Figures and Tables

**Table 1:** Dark- and photo- (1 J/cm^2^) cytotoxicity of amino acid conjugates of chlorin-e_6_ in Hep2 cells.

Compound	Phototoxicity (IC_50_, μM)	Dark Toxicity (IC_50_, μM)
Chlorin e_6_ (**14)**	20.8	>400
17^3^-LysChlorin e_6_TME	26.2	>400
15^2^-AspChlorin e_6_DME	4.0	373.1
15^2^-LysChlorin e_6_TME	28.8	>400
15^2^-AspPdChlorin e_6_DME	16.7	324.8
15^2^-LysPdChlorin e_6_TME	3.3	>400
13^1^-AspChlorin e_6_DME	0.6	284.6
13^1^-βAlaAspChlorin e_6_DME	0.8	383.9
13^1^-EDLysChlorin e_6_DME ^1^	1.3	268.4
13^1^-LysChlorin e_6_TME	0.6	34

^1^ ED: ethylenediamine.

## Data Availability

Not applicable.
